# Impact of AI-aided colonoscopy in clinical practice: a prospective randomised controlled trial

**DOI:** 10.1136/bmjgast-2023-001247

**Published:** 2024-01-30

**Authors:** Johanna Schöler, Marko Alavanja, Thomas de Lange, Shunsuke Yamamoto, Per Hedenström, Jonas Varkey

**Affiliations:** 1 Medical Department, Sahlgrenska University Hospital, Goteborg, Sweden; 2 Department of Molecular and Clinical Medicine, Institute of Medicine, University of Gothenburg, Goteborg, Sweden; 3 Department of Medicine, Sahlgrenska University Hospital, Goteborg, Sweden; 4 Department of Gastroenterology and Hepatology, National Hospital Organization Osaka National Hospital, Osaka, Japan; 5 Division of Gastroenterology, Department of Medicine, Sahlgrenska University Hospital, Gothenburg, Sweden

**Keywords:** COLONOSCOPY, COLONIC ADENOMAS, COLORECTAL CANCER, COLONIC POLYPS

## Abstract

**Objective:**

Colorectal cancer (CRC) has a significant role in cancer-related mortality. Colonoscopy, combined with adenoma removal, has proven effective in reducing CRC incidence. However, suboptimal colonoscopy quality often leads to missed polyps. The impact of artificial intelligence (AI) on adenoma and polyp detection rate (ADR, PDR) is yet to be established.

**Design:**

We conducted a randomised controlled trial at Sahlgrenska University Hospital in Sweden. Patients underwent colonoscopy with or without the assistance of AI (AI-C or conventional colonoscopy (CC)). Examinations were performed with two different AI systems, that is, Fujifilm CADEye and Medtronic GI Genius. The primary outcome was ADR.

**Results:**

Among 286 patients, 240 underwent analysis (average age: 66 years). The ADR was 42% for all patients, and no significant difference emerged between AI-C and CC groups (41% vs 43%). The overall PDR was 61%, with a trend towards higher PDR in the AI-C group. Subgroup analysis revealed higher detection rates for sessile serrated lesions (SSL) with AI assistance (AI-C 22%, CC 11%, p=0.004). No difference was noticed in the detection of polyps or adenomas per colonoscopy. Examinations were most often performed by experienced endoscopists, 78% (n=86 AI-C, 100 CC).

**Conclusion:**

Amidst the ongoing AI integration, ADR did not improve with AI. Particularly noteworthy is the enhanced detection rates for SSL by AI assistance, especially since they pose a risk for postcolonoscopy CRC. The integration of AI into standard colonoscopy practice warrants further investigation and the development of improved software might be necessary before enforcing its mandatory implementation.

**Trial registration number:**

NCT05178095.

**WHAT IS ALREADY KNOWN ON THIS TOPIC**

- Colorectal cancer continues to be the second most significant contributor to cancer-related deaths. There is a clear link between the detection of adenoma and sessile serrated lesions with the reduction of postcolonoscopy colorectal cancer. However, the extent of artificial intelligence (AI) influence on polyp detection in real-life situations is still in the process of being definitively determined.

**WHAT THIS STUDY ADDS**

- This study presents a novel randomised controlled trial. While AI did not demonstrate improvements in adenoma detection in clinical practice, it was observed to increase detection rates for sessile serrated lesions.

**HOW THIS STUDY MIGHT AFFECT RESEARCH, PRACTICE OR POLICY**

- Our findings suggest that the integration of AI into standard practice warrants further investigation. Additionally, enhancing the software might be essential before considering its mandatory integration. Nevertheless, improved detection of sessile serrated lesions may prove important to prevent colorectal cancer.

## Introduction

 Colorectal cancer (CRC) remains the second-leading cause of cancer-related mortality.^1^ Efficacious colonoscopy coupled with adenoma removal has demonstrated a reduction in CRC incidence and mortality.^2^ The adenoma detection rate (ADR) and the detection of sessile serrated lesions (SSL) exhibits a direct correlation with reducing postcolonoscopy CRC occurrences.^3^,^5^ However, the suboptimal quality of numerous colonoscopies has led to the oversight of a substantial number of polyps, underscoring the significance of enhanced visualisation.^6 7^

The introduction of key performance indicators, innovative imaging methodologies and novel endoscopic technologies has been pursued in recent years to improve ADR.^8^ Moreover, the improvements in machine learning and deep learning have facilitated the development of numerous artificial intelligence (AI) softwares, aimed at further refining polyp detection, as concisely summarised by Hoerter *et al*.^9^ Computer-aided detection (CADe) systems, using AI algorithms, have been validated for their efficacy in polyp detection during colonoscopy.^10^ These CADe systems are now commercially accessible and have seamlessly integrated into routine endoscopy procedures across multiple centres.

However, the impact of AI on polyp detection in real-world scenarios is yet to be fully established. Some studies show an augmentation in polyp and ADRs during colonoscopy, particularly for smaller polyps, when CADe is used as opposed to conventional colonoscopy (CC) techniques.^11 12^ Nevertheless, a recent real-world investigation demonstrated a notable decrease in ADR and polyp detection rate (PDRs) following the implementation of an AI system.^13^ Remarkably, the Danish Health Technology Council has recently released a directive cautioning against the adoption of AI in Colonoscopy, attributing potential complications and overtreatment arising from the polypectomy of minute polyps.^14^

The current data available is limited regarding the influence of CADe on colonoscopy performance based on the endoscopist’s expertise. The future role of AI in routine colonoscopy remains somewhat uncertain. Notably, the European Society of Gastrointestinal Endoscopy has recently disseminated a position statement delineating the anticipated value of AI in endoscopy, stipulating that AI should be used to elevate the ADR of less experienced endoscopists to the calibre of their proficient counterparts.^15^

This study seeks to investigate the potential improvement of ADR and PDR through the implementation of two distinct AI systems, namely GI Genius by Medtronic and Fujifilm CADEYE. This study was conducted in a real-world clinical setting at two hospitals in Sweden.

## Methods

This prospective, randomised controlled trial was conducted at the endoscopy centres of Sahlgrenska University Hospital, that is, Sahlgrenska Hospital and Mölndal Hospital in Sweden. Patients were enrolled between 31 August 2020 and 6 April 2022. This was an investigator-initiated study. The study protocol was prospectively registered on ClinicalTrials.gov (NCT05178095).

### Study population

We included adult patients between the age interval of 40 and 90 years. Patients with a history of inflammatory bowel disease (IBD), contraindication for polypectomy or known polyps were excluded. Incomplete examinations due to factors such as obstructive cancer, technical issues or inadequate bowel preparation, as well as cases where the Boston Bowel Preparation Scale (BBPS)^16^ <2 in 1 segment or a total BBPS<6, were excluded from the primary analysis. Indications for performing the examination included cancer screening, alarm symptoms (ie, iron-deficiency anaemia, suspicion of malignancy following rectal examination and CT findings that raise suspicion of malignancy), inconclusive CT findings (suggestive of a benign but inconclusive cause) and other (positive faecal occult blood stool test, polyp surveillance, hereditary CRC, diarrhoea).

### Randomisation

Patients were randomly assigned to either undergo AI-assisted colonoscopy (AI-C) or without AI, that is, conventional colonoscopy (CC) in a 1:1 ratio. Sealed envelopes in blocks of four were used for randomisation. The endoscopist and patient were not blinded, as the endoscopist could notice if AI was activated during the colonoscopy. The colonoscope insertion followed clinical routine, with AI being deactivated during intubation and activated during withdrawal in the AI-C group. In the CC group, the AI system remained deactivated throughout the procedure. Exclusions were made due to unclean bowel or inability to complete the examination by the endoscopist. Pathology specimens were assessed by pathologists blinded regarding study group allocation.

### Colonoscopy procedure and data collection

Before study patients were included, all endoscopists underwent comprehensive training in the AI systems, including pilot procedures during a run-in phase. The study sites used Evis X1, Olympus and Medtronic GI Genius at Mölndal Hospital and Fujifilm (EC760 series) with CAD EYE at Sahlgrenska Hospital for endoscopy and AI systems. The selection of the AI system was not based on stratification but rather on practical considerations. Sahlgrenska Hospital had Fujisystems in place, which is a prerequisite for CAD EYE. Standard or paediatric high-definition colonoscopes were used. When the AI system detected a suspected lesion, it marked the lesion on the monitor with a rectangular bounding box. Fujifilm CAD EYE additionally enabled polyp characterisation as neoplastic or hyperplastic. Sedation, a combination of benzodiazepines and opioids, was administered at the endoscopist’s discretion. Bowel preparation involved 4 L of polyethylene glycol (PEG) in split dose or sodiumpicosulfate. Colon cleanliness was assessed using BPPS. Following caecal intubation, withdrawal time was measured by the assistant and stopped by the end of the procedure, withdrawal time included therapeutic procedures. Withdrawal was performed using either white light imaging or linked colour imaging (LCI), depending on the preference of the examiner. Polyp morphology was evaluated using the Paris^17^ and NBI International Colorectal Endoscopic classification (NICE)^18^ . Polyp size, location, polypectomy method and other endoscopic findings were recorded. Adverse events following the procedure were also documented. Both experienced and inexperienced endoscopists participated, with inexperienced defined as those who had conducted fewer than 400 colonoscopies.

### Outcomes

The primary outcome was ADR in each arm, defined as the number of patients with at least one histologically confirmed adenoma divided by the total number of colonoscopies performed. Secondary outcomes included the PDR, the number of adenomas and polyps per colonoscopy (APC and PPC, respectively), the number of SSL per colonoscopy (SSLPC), the SSL detection rate (SSLDR), the proportion of polyps smaller than 5 mm, the proportion of examinations with polyps located in the right colon and a comparison of ADR, PDR, SSLDR and the presence of polyps smaller than 5 mm in both experienced and inexperienced endoscopists separately when comparing CC to AI-C. Additionally, ADR and SSLDR were calculated for CAD-EYE and GI Genius.

### Sample size estimation and statistical analysis

A two-tailed sample size calculation for non-paired samples with a dichotomous outcome was performed to detect a difference in ADR of 17% comparing AI-C with CC. This was based on the prevalence of ADR in a large Swedish colon cancer screening cohort, that is, 14%^19^ and a detection rate of 39% from a recent AI study,^20^ thus leaving a safety margin. With a type 1 error set at 0.05 and statistical power at 80%, a total sample size of 240 cases (120 per arm) was determined.

Descriptive statistics were reported as mean with SD for continuous variables and percentage for categorical variables. The mean value, that is, for procedure-related factors: patient age, time spent per colonoscopy, withdrawal time and total number of PPC between groups were compared with Student’s t-test. The cleanliness of bowel preparation, assessed using the BPPS scale, was compared using the Mann-Whitney U test. Fisher’s exact test was used to compare PDR, ADR and SSLDR, patient sex, indication for the examination and given medication. The impact of endoscopist experience on polyp and adenoma detection was analysed by comparing experienced and inexperienced endoscopists (AI-C vs CC). A p<0.05 was considered statistically significant. Statistical analyses were performed with SPSS V.26.0 (SPSS). CIs for relative risk were calculated using the Wald approximation using the R-package epitools.

## Results

### Study population

**Figure 1:**
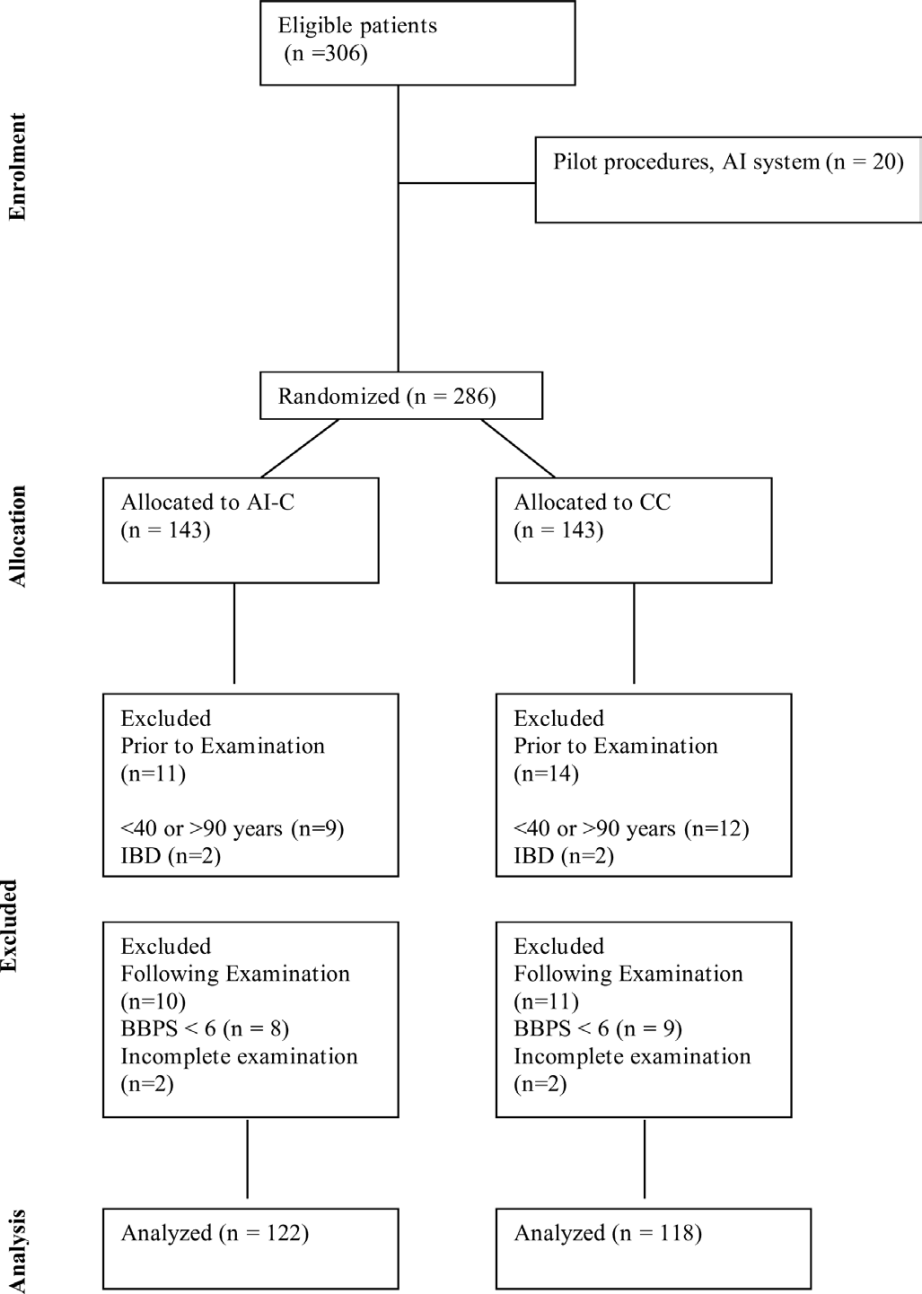
Consolidated Standards of Reporting Trials (CONSORT) study flow chart. AI, artificial intelligence; AI-C, AI-assisted colonoscopy; BBPS, Boston Bowel Preparation Scale; CC, conventional colonoscopy; IBD, inflammatory bowel disease.

A total of 306 patients were eligible, 20 patients were available for the pilot procedures but were not included in the study. A total of 286 patients were randomised into two groups: AI-C or CC. Prior to examination, 25 patients were excluded due to exclusion criteria such as known IBD or age criteria (<40 or >90 years). Among the remaining 261 patients, 21 were subsequently excluded after the examination: some due to failure to reach the cecum, and others due to inadequate bowel cleansing (BBPS<6). Consequently, 240 patients were included in the final analysis (figure 1).

Colonoscopies were conducted at two endoscopy units within Sahlgrenska University Hospital: 47 (20%) at the Mölndal Hospital and 193 (80%) at the Sahlgrenska Hospital. The mean age (SD) of the study participants was 66.4 years (±11.5), with comparable distributions across both the AI-C and CC groups. Gender distribution was uniform, with an equal representation of male and female patients in each group. The indications for colonoscopy were alarm symptoms (56%), other indications including positive occult blood tests, polyp or cancer surveillance, and diarrhoea (40%), and a minority of patients were scheduled for colonoscopy following findings on a CT scan (3%). Organised cancer screening was only initiated towards the end of the study period, thereby accounting for the low proportion (1%) of patients undergoing colonoscopy for screening purposes (table 1).

**Table 1:** Clinical characteristics of the patients

Characteristics	Total	AI-C	CC	P value
Age, years (SD)	66.4 (11.5)	65.9 (11.5)	66.8 (11.5)	0.52
Sex, no male (%)	120 (50)	64 (53)	57 (47)	0.52
Procedure indication				
Cancer screening	2 (1)	1 (1)	1 (1)	1
Alarm symptoms	134 (56)	71 (58)	63 (53)	0.52
Inconclusive CT findings	8 (3)	2 (2)	6 (5)	0.17
Other (positive FOBT, polyp surveillance, hereditary colorectal cancer, diarrhoea)	96 (40)	48 (39)	48 (41)	0.89
Site				
>Sahlgrenska Hospital, no (%)	193 (80)	98	95	
>Mölndal Hospital, no (%)	47 (20)	24	23	

AI-C, artificial intelligence-assisted colonoscopy; CC, conventional colonoscopy; Positive FOBT, positive faecal occult blood stool test.

### Intraprocedural characteristics

The procedure-related data, as outlined in table 2, reveal no significant discrepancies between the AI-C and the CC groups in terms of bowel cleanliness, bowel preparation regimen, insertion depth, usage of a cap and administered anaesthetics. Nearly all examinations (94%) exhibited a withdrawal time exceeding 6 min. Notably, the total withdrawal time was found to be significantly prolonged in the AI-C group (21.4 min±13.5), in comparison to the CC group (17.4 min±13.1) p=0.03. Endoscopists were encouraged to use the image enhancing technique LCI along with AI, when using Fujifilm endoscopes, resulting in a higher prevalence of LCI usage in the AI group (11% in the CC group compared with 45% in the AI-C group, p=0.0). Cap utilisation was observed in 85 patients (35%) across both groups, exhibiting no significant variance.

**Table 2:** Procedure-related data

Procedure-related data	Total	AI-C	CC	P value
BBPS	8.3 (1.2)	8.2 (1.2)	8.3 (1.1)	0.79
Insertion level				
Cecum	63 (26%)	32 (26%)	31 (26%)	
Terminal ileum	177 (74%)	90 (74%)	87 (74%)	
Withdrawal times in minutes				
Including intervention	19.4 (13.4)	21.4 (13.5)	17.4 (13.1)	0.03
Without intervention	13 (8.4)	13.9 (7.3)	12.3 (9.4)	0.98
Withdrawal times in categories				
0–5 min	3 (1%)	1 (1%)	2 (2%)	
6–9 min	33 (14%)	11 (10%)	22 (19%)	
>10 min	190 (80%)	100 (89%)	90 (79%)	
Missing data	14 (6%)			
Used image enhancing technology				
White light	172 (72%)	67 (55%)	105 (89%)	
Linked colour imaging	67 (28%)	54 (45%)	13 (11%)	<0.001
Use of cap	85 (35%)	51 (46%)	34 (34%)	0.08
Bowel preparation				
PEG	209 (87%)	105 (86%)	104 (88%)	
Sodium picosulphate	8 (3%)	5 (4%)	3 (2.5%)	
Moviprep	6 (3%)	3 (3%)	3 (2.5%)	
Other, missing	17 (7%)	9 (7%)	8 (7%)	
Anaesthesia				
Opioid	174 (75%)	94 (80%)	80 (70%)	
Benzodiazepine	189 (79%)	96 (81%)	93 (82%)	
General anaesthesia	0	0	0	
Endoscopist experience				
Experienced	186 (78%)	86 (70%)	100 (85%)	
Inexperienced	54 (22%)	36 (30%)	18 (15%)	

Data presented as number (%), mean (SD).

AI-C, artificial intelligence-assisted colonoscopy; BPPS, Boston Bowel Prep Score; CC, conventional colonoscopy; PEG, polyethylene glycol.

#### Adverse events

There were no immediate adverse events in either the AI-C or CC group.

### Outcome measures

The results of the outcome measures are summarised in table 3. The ADR, representing the primary outcome, was 42% for all patients and there was no significant difference between patients in the CC group and the AI-C group (41% vs 43%, p=0.70). The overall PDR reached 61%, with 57% in the CC group and 65% in the AI-C group (p=0.24). No discernible variation was noted in the detection rate of malignant lesions (8 patients in CC group vs 5 patients in the AI-C group, p=0.4).

**Table 3:** Outcome measures

Outcome measures	AI-C	CC	RR (95% CI)	P value
Detection rate				
ADR	53 (43%)	48 (41%)	1.07 (0.79 to 1.44)	0.696
PDR	79 (65%)	67 (57%)	1.14 (0.93 to 1.40)	0.235
PDR (<5 mm)	50 (41%)	43 (36%)	1.12 (0.82 to 1.55)	0.509
SSLDR	27 (22%)	13 (11%)	2.0 (1.09 to 3.70)	0.024
Right-sided polyps*	59 (48%)	51 (43%)	1.12 (0.85 to 1.48)	0.44
Right-sided polyps (not SSL)	37 (39%)	38 (36%)	0.94 (0.65 to 1.37)	0.77
Cancer	5 (4%)	8 (7%)	0.6 (0.2 to 1.8)	0.404
Hyperplastic polyps	9 (7%)	6 (5%)	1.45 (0.53 to 3.95)	0.6
Polyps per colonoscopy				
All polyps	2.0 (2.6)	1.5 (1.6)		0.1
Adenomas	0.89 (1.3)	0.72 (1.1)		0.3
Right-sided Polyps	1.34 (1.3)	1.24 (1.0)		0.6
Polyps <5 mm	0.66 (1.0)	0.53 (0.9)		0.3
SSL	0.3 (0.7)	0.17 (0.5)		0.1
Inexperienced endoscopists	36 (67%)	18 (33%)		
ADR	15 (42%)	5 (27%)	1.50 (0.65 to 3.47)	0.4
PDR	19 (53%)	9 (50%)	1.06 (0.61 to 1.84)	1.0
Right-sided polyps*	11 (31%)	6 (33%)	0.92 (0.40 to 2.08)	1.0
Polyps <5 mm	10 (28%)	6 (33%)	0.83 (0.36 to 1.93)	0.8
SSLDR	4 (11%)	2 (11%)	1.0 (0.2 to 5.0)	1.0
Experienced endoscopists	86	100 (54%)		
ADR	38 (44%)	43 (43%)	1.03 (0.74 to 1.43)	0.9
PDR	60 (70%)	58 (58%)	1.20 (0.97 to 1.49)	0.1
Right-sided polyps*	48 (56%)	45 (45%)	1.24 (0.93 to 1.65)	0.2
Polyps <5 mm	40 (47%)	37 (37%)	1.26 (0.89 to 1.77)	0.2
SSLDR	23 (27%)	11 (11%)	2.4 (1.3 to 4.7)	0.01

Data presented as number (%), mean (SD).

* Detection rate of right-sided polyps.

ADR, adenoma detection rate; AI, artificial intelligence; CC, conventional colonoscopy; PDR, polyp detection rate; RR, relative risk; SSLDR, sessile serrated lesions detection rate.

Subgroup analyses were conducted to examine the effects on specific polyp types, SSLDR was higher in the AI-C group (22%) compared with the CC group (11%), p=0.02. The detection rate of small polyps (<5 mm) was comparable in both groups (36% in the CC group, 41% in AI-C group, p=0.5). Regarding polyp localisation, no difference was observed in the detection of right-sided polyps compared with polyps distal of the splenic flexure based on AI utilisation (43% in the CC group and 48% in the AI-C group, p=0.4).

The number of PPC was 1.7 (SD 2.2) and the number of APC was 0.8 (SD 2.2). Neither PPC nor APC differed significantly between the two groups (see table 3).

When comparing the performance of each system, CAD EYE exhibited an ADR of 47% (46/98), whereas GI Genius had an ADR of 29% (7/24), with a p value of 0.168. In the CAD EYE control group, the ADR was 45% (43/95), compared with the control group of GI Genius, which had an ADR of 22% (5/23), resulting in a p value of 0.057. The SSLDR was 18% (18/98) for CAD EYE, as opposed to 38% (9/24) for GI Genius, with a p value of 0.056. In the control group for CAD EYE, the SSLDR was 13% (12/95), while GI Genius had a rate of 5% (1/22), yielding a p value of 0.3.

### Experienced and inexperienced endoscopists

The stratification of endoscopists based on experience levels yielded no significant advantage for AI-C within either group (table 3). Nevertheless, there was a numerical discrepancy, ADR for less experienced endoscopists showing 27% in CC and 42% in AI-C (p=0.4). Among experienced endoscopists, the ADR reached 43% in CC and 44% with AI-C, respectively (p=0.9).

When examining the SSLDR, a significant disparity became evident within the experienced group (11% CC and 27% with AI-C, p=0.01), while no such distinction manifested within the inexperienced group (11% in both AI-C and CC group).

## Discussion

In this prospective randomised study, AI-C did not lead to significant improvement in ADR, ADR or the number of APC and PPC, when compared with CC. However, we observed a higher detection rate of SSL in the AI-C group. Concerning small polyps <5 mm and right-sided polyps, there was no significant benefit from AI.

### Adenoma detection rate

The impact of AI on ADR in our study does not align perfectly with previous studies and meta-analyses. In an Italian multicentre study with a similar design to ours, the authors noted an ADR improvement with CADe from 40% to 54%.^21^ The improvement was primarily due to a higher detection rate of small polyps measuring <9 mm. Similar findings have been demonstrated by other authors.^11^ The ADR of 42% in our study surpasses the internationally recommended standard for high-quality endoscopy, with an ADR over 25%,^22^ and it even exceeds the average ADR in our country as reported by the Swedish National Sweden Registry for Colonoscopies,^23^ which indicated an ADR of 37% for the year 2021. The relatively high ADR, which remained high even in the CC group, might help explain the lack of additional benefits observed from the implementation of AI. Additionally, our study predominantly used a different AI system compared with the Italian study.

The withdrawal time of 12.3 min in the CC group and 13.9 min in the AI-C group, was markedly prolonged significantly surpassing the recommended minimum duration of >6 min, for a diagnostic high-quality endoscopy. The possibility that participating endoscopists were influenced cannot be disregarded, leading to a heightened level of scrutiny compared with their usual practice, given the awareness of their involvement in a scientific study. Furthermore, the withdrawal time did not significantly differ between the two groups after adjusting for therapeutic interventions. This contrasts with findings from previous studies.^11^ Several additional factors might account for the elevated ADR observed in both groups. Bowel cleanliness met the quality criteria for all patients, as those with BBPS<6 were excluded from the study. A majority of the endoscopists were experienced in these procedures. Routine utilisation of high-definition instruments, coupled with the frequent application of technical and optical enhancement tools like LC and the use of a cap. These circumstances imply that conditions were well-optimised for polyp detection, even within the group not using AI.

### SSL detection rate

To the best of our knowledge, the improved SSLDR with AI has not previously been described. This finding holds particular interest, especially due to a recent study that analysed the detection rate of right-sided serrated polyps. This study revealed that even a modest 1% increase in detection rate resulted in a substantial 7% reduction in the risk of interval CRC.^24^ An additional potential explanation for the high SSLDR in the AI-C group, aside from CADe-usage, might be the more frequent application of LCI. LCI is an optical enhancement technique designed to highlight red and white colours, developed to enhance the visibility of flat lesions. Notably, several studies indicate that LCI usage point towards a better detection rate of flat lesions and SSL.^25 26^ However, a similar trend was noted in the GI Genius group where Olympus instruments were used, perhaps emphasising the importance of the AI system itself.

Overall, the SSLDR was elevated in both groups (11% in the CC group and 22% in the AI-C group), compared with previous results showing detection rates of 2% for SSL.^27^ A potential explanation for this difference in detection rate is the heterogeneity in the definitions of SSL. The recently established nomenclature by WHO (2019) for serrated lesions refers to SSL for lesions that have previously been called both sessile serrated adenomas and sessile serrated polyps.^28^

It has been described that AI can assist the endoscopists in detecting challenging lesions, including diminutive polyps and those situated at the periphery of the endoscopic field.^29^ SSL, characterised by their flatter and paler appearance compared with other polyps, sometimes become concealed by a layer of mucus,^30^ rendering them more difficult for endoscopists to detect. This challenge highlights the potential benefit of AI in such scenarios.

### Experienced versus inexperienced

The usage of a CADe system resulted in an improvement on ADR for inexperienced endoscopists, although not statistically significant. A similar improvement was not detected among the more experienced endoscopists. Notably, the number of inexperienced participants was relatively limited (N=54). In a study by Ainechi *et al*,^31^ inexperienced endoscopists viewed short colonoscopy videos both with and without an AI system, revealing a significant benefit with the inclusion of an AI system.^31^ However, it is unclear how these results apply in a real-life setting. Interestingly, a tandem study from China demonstrated a decrease in adenoma missed rates among inexperienced endoscopists using AI, thereby making them non-inferior to experts and indicating an improvement in ADR.^32^ This suggests that while CADe systems can contribute to raising the ADR, current evidence does not support a specific recommendation based on endoscopy experience level. Nevertheless, our own data support additional advantages for inexperienced endoscopists.

### Limitations

We were able to investigate the effect of CADe in a diverse group of patients within a real-world setting, which to our knowledge has not been previously demonstrated in a Scandinavian cohort, gaining a deeper understanding of the advantages of AI in a clinical context. Nevertheless, it is important to acknowledge certain limitations. The extended duration of our project, predominantly caused by the pandemic, coupled with the ongoing evolution of AI systems during this timeframe, and the use of two different systems (Fujifilm and Medtronic) at two different sites, might have influenced the outcome.

In conclusion, our study assessed the clinical impact of CADe in clinical practice. In this setting, our findings suggest that AI did not significantly improve ADR. However, AI appears promising in the detection of challenging lesions such as SSL. Further research is needed to confirm this finding.

## Data Availability

Data are available on reasonable request.
